# Impact of Early Ying Yang Bao Nutritional Support on Growth and Neurodevelopment in Preschool Children in China

**DOI:** 10.3390/nu16223906

**Published:** 2024-11-15

**Authors:** Xiayu Zhao, Tingting Liu, Chao Han, Jinpeng Zhao, Yan Li, Junsheng Huo, Qin Zhuo, Zhaolong Gong

**Affiliations:** Key Laboratory of Trace Element Nutrition of National Health Commission, National Institute for Nutrition and Health, Chinese Center for Disease Control and Prevention, Beijing 100050, China; zhaoxy@ninh.chinacdc.cn (X.Z.); liutt@ninh.chinacdc.cn (T.L.); hanchao@ninh.chinacdc.cn (C.H.); zhaojp@ninh.chinacdc.cn (J.Z.); liyan@ninh.chinacdc.cn (Y.L.); huojs@ninh.chinacdc.cn (J.H.); zhuoqin@ninh.chinacdc.cn (Q.Z.)

**Keywords:** preschool children, growth, neurodevelopment, Ying Yang Bao

## Abstract

**Background**: Providing early nutritional support through Ying Yang Bao (YYB) can assist children in achieving their full developmental potential. We aimed to examine the lasting impact of YYB and how growth affects neurodevelopment in preschool children. **Methods**: 1104 children aged 1 year were divided into a YYB control group (YYB-CG) and a YYB intervention group (YYB-IG). Information on basic characteristics, anthropometric measurements, dietary status, YYB consumption, and neurodevelopment for these children was taken annually from 2018 to 2022 until they reached 5 years old. Confounders were well balanced using propensity score matching (PSM), and then 474 pairs of children were included in subsequent analyses. The comparison between groups was performed using *t*-tests or chi-square analyses. Linear regressions were used to examine the independent associations between children’s dimensions (Z-score for weight relative to age [WAZ], Z-score for height relative to age [HAZ], Z-score for body mass index by age [BAZ], and conditional measures of height- and weight-based growth) and neurodevelopment. **Results**: Children in the YYB-IG had higher scores in the mental index (MI), the developmental quotient (DQ), height, and BAZ (*p* < 0.05) and had a lower risk of stunting. Accelerated weight gain from ages 1 to 5 (β (95% confidence interval [CI]): 0.26 (0.08–0.45)) and increased height gain during this period (β (95% CI): 0.68 (0.14–1.23)) were associated with greater MI. A higher WAZ was linked to increased MI at 1 year (β (95% CI): 0.89 (0.09–1.68)), 2 years (β (95% CI): 0.99 (0.20–1.78)), 3 years (β (95% CI): 0.92 (0.15–1.69)), 4 years (β (95% CI): 0.88 (0.09–1.68)), and 5 years of age (β (95% CI): 1.01 (0.28–1.74)). An increased HAZ corresponded with a higher MI score at ages 1 year (β (95% CI): 1.47 (0.75–2.20)), 2 years (β (95% CI): 1.25 (0.49–2.02)), 3 years (β (95% CI): 1.11 (0.31–1.90)), 4 years (β (95% CI): 0.93 (0.12–1.74)), and 5 years old (β (95% CI): 1.17 (0.43–1.90)); higher DQ levels were also recorded at 1 year (β (95% CI): 0.82 (0.10–1.55)) and 5 years of age (β (95% CI): 0.79 (0.06–1.51)). **Conclusions**: YYB can improve specific areas of neurodevelopment and growth in preschool children. Additionally, children’s linear growth is positively linked to neurodevelopment in those of preschool age.

## 1. Introduction

Early life serves as a critical window for developing human capital and an essential period for interventions aimed at improving children’s nutritional status throughout their lifetimes. Despite growing global dedication and increased assistance, poor early developmental progress remains a major public health issue, with around 317 million children not reaching their optimal developmental potential [1]. Also, one-third of children and adolescents worldwide exhibit poor growth outcomes, with 22.3% affected by stunting and 6.8% classified as underweight [2]. Children aged 6–23 months are considered a vulnerable population. In rural China, infants between 6 and 23 months old have the highest rates of malnutrition among children under the age of 5 years [3]. Malnutrition during this crucial period results in significant growth delays, and inadequate development during the first 1000 days of life has lasting negative effects on future educational outcomes and other aspects of human development [4]. To address child malnutrition, the Ministry of Health of China has implemented initiatives aimed at enhancing nutritional practices. Following years of research by Chinese nutrition experts, a nutrition pack known as YYB was developed. In 2012, the Ministry of Health and the All-China Women’s Federation launched the Nutrition Improvement Project for Children in Impoverished Regions (NIPCPAC). This initiative supplied 9.47 million infants aged 6–24 months across 21 provinces in central and western China with a daily free package of YYB to combat child malnutrition [5].

YYB is a home-based, fortified, complementary food, as detailed in our earlier publications [5,6,7]. To summarize, YYB is made from soybean or milk powder enriched with a range of nutrients and additional ingredients, like protein, calcium, iron, zinc, folic acid, and vitamins A, C, and D, all adhering to national food safety regulations (Dietary Supplements GB 22570-2014) [6]. Numerous studies in China have indicated that an 18-month YYB intervention can significantly decrease malnutrition in children aged 6–24 months [8,9]. A meta-analysis conducted by the project team of the Nutrition Research Institute of the Chinese Center for Disease Control and Prevention showed that children aged 6 to 24 months who receive YYB for 18 months experienced a significant reduction in malnutrition rates [10]. In 2018, the Institute of Nutrition and Health at the Chinese Center for Disease Control and Prevention initiated the Long-term Health Effects Assessment Project for Infants and Toddlers Nutrition Pack (LHEAPITNP) to monitor the nutritional status of children aged 24 months and older. This project is a prospective, controlled study designed to investigate both the immediate and long-term impacts of YYB on the nutritional status of children.

Many diets lack essential nutrients that are crucial for brain development, often resulting in imbalances [11]. Providing supplements containing iron, folic acid, and other nutrients during the preschool period is a frequently suggested approach for enhancing child health results, particularly in undernourished groups [12]. Although evidence reinforces the significance concerning nutrition throughout the preschool stage for children’s wellness and progress [13], data from trials involving humans is limited, and the impacts of YYB on neurodevelopment in early childhood and beyond remain largely unclear. In addition, evidence regarding the relationship between early growth and neurodevelopment or educational achievement is limited and inconsistent. A birth cohort study in New Delhi, India, found that children’s linear growth at an age of 3 years positively correlated with cognitive ability, with body shape and growth measurements accounting for 8% of education attainment (one indicator of cognitive ability) [14]. Conversely, Upadhyay et al. [15] did not observe these relationships in a large study of Indian children aged 1–3 years.

We have a special chance to fill important gaps in this discussion by observing the long-term effects of YYB among preschool children to understand how the timing of growth affects neurodevelopment in children using a longitudinal birth cohort study of early childhood interventions in China.

## 2. Materials and Methods

### 2.1. Trial Design and Children

**Figure 1:**
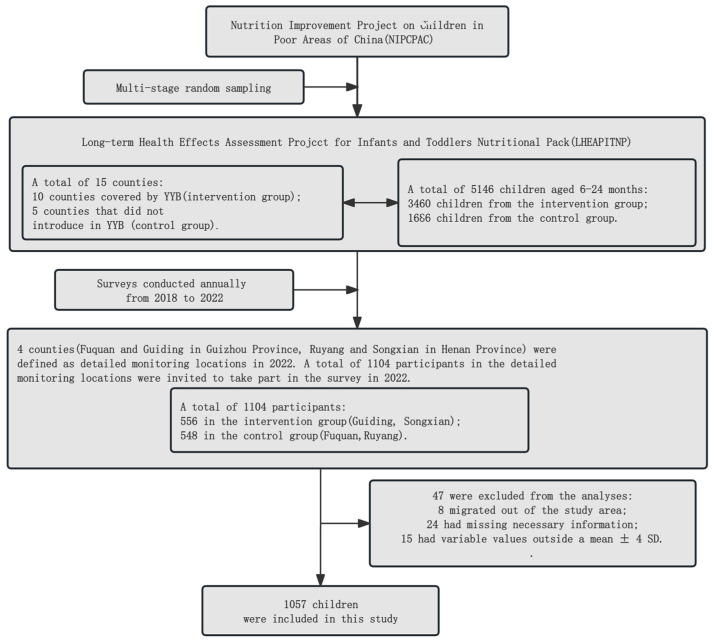
Procedure for enrolling eligible participants in the current study.

Data for the study were gathered from the LHEAPITNP between 2018 and 2022. As outlined in our earlier publication, this study employed multi-stage sampling, probability proportional to size, and random equidistant sampling methods to select the samples [16]. For follow-up monitoring, this study chose two intervention counties (Guiding and Songxian) and two control counties with comparable geographic and economic conditions (Fuquan and Ruyang). About 300 children were randomly chosen from each of the sample counties. For our study, we focused on the average age to describe and analyze the characteristics of children: 1 year in 2018 (1.25 ± 0.42), 2 years in 2019 (2.18 ± 0.42), 3 years in 2020 (3.22 ± 0.42), 4 years in 2021 (4.14 ± 0.43), and 5 years in 2022 (5.14 ± 0.42). As shown in Figure 1, we excluded participants who migrated out of the study area, those with missing necessary information, and outliers, defined by those exceeding the mean ± 4 standard deviations (SDs). To effectively reduce confounding effects, PSM was conducted using baseline information for a more accurate comparison between the groups [17]. After quality control, the final analytical sample included 948 children with valid data. These participants’ basic information was all available, including anthropometric data from 2018 to 2022 and neurodevelopment outcomes measured in 2022.

### 2.2. Outcome Measures

The general neurodevelopmental outcome of children was assessed using the Chinese Improved Developmental Screen Test—Standard Edition (CIDST-S), which is used for assessing the cognitive abilities of children under 6 years old [18]. The CIDST-S comprises 120 core subtests, categorized into three zones—30 subtests for the physical fitness zone, 30 for social adaptation, and 60 for intellectual fitness. These subsets are aggregated into two indices representing neurodevelopment across two specific cognitive domains, including the MI and DQ. Validation and adaptation of the test for the Chinese context have been completed, including translation, cultural review, alterations, and standardization [18]. Standardization results indicated that interrater reliability was high, with coefficients exceeding 90% for the CIDST-S subsets [18]. The CIDST-S was administered at professional training venues by well-trained researchers, including pediatricians and individuals holding a master’s degree in public health. These researchers underwent 1 week of comprehensive training conducted by experts from the China CDC Nutrition Institute, featuring educational lectures, discussions, role-playing interviews, field experience, and debriefing activities. To maintain assessment quality, weekly oversight in the field and monthly meetings with staff were carried out, along with site visits by the study investigators. Additionally, annual refresher training sessions took place following the initial training to maintain standardized testing.

Children’s anthropometric data were recorded at various intervals by trained field workers using standardized methods [19]. The children’s weight was measured using electronic scales calibrated to 10 g, with daily accuracy checks against standard weights. The children’s length in a lying position was recorded using collapsible length boards with a precision of 1 mm. The measurements were then converted to HAZs, WAZs, and BAZs using the World Health Organization (WHO) Anthro software in line (https://www.who.int/tools/child-growth-standards/software; accessed on 10 November 2023) with the 2006 WHO Child Growth Standards [20]. HAZ, WAZ, and BAZ values below −2 were used to define stunting, underweight, and wasting, respectively.

To assess the significance of growth between ages 1 and 5, we considered two primary indicators: (1) the child’s attained size at ages 1–5 (including weight, height, WAZ, HAZ, and BAZ) and (2) linear and ponderal growth measurements conditioned for ages 1 to 5, which were considered significant exposure variables. Given the strong correlation between linear growth and weight gain, along with repeated measures for each child, we established conditional growth indicators to generate independent height and weight gains for the 1- to 5-year period. Standardized residuals from linear regressions of current anthropometric measurements (i.e., at age 5) on earlier data (i.e., from age 1) were used to calculate conditional relative height and weight gains between ages 1 and 5. Specifically, conditional height gain reflects the current height adjusted for height and weight at age 1, while conditional relative weight gain reflects the current weight modified by current length and all prior weight and length measurements. Conditional relative height and weight gains indicate deviations from each child’s prior growth path, representing the relative rates of length and weight gain throughout this developmental stage. This methodology has been extensively utilized in prior research [21,22], making it possible to compare the influences of linear growth and weight gain relative to outcomes.

### 2.3. Other Variables

The detailed sampling procedure was described in our previous study [16]. Questionnaires designed by NIPCPAC experts were used to collect data on the essential characteristics of children, including questionnaire investigations and physical examinations. Drawing from the LHEAPITNP framework and prior research [23,24], we incorporated data on various factors, including children’s basic information (such as age, gender, birth weight, birth length, and birth outcomes, like preterm delivery (<37 weeks) [25]), caregiver characteristics (parent–child care, education level, and economic status), and dietary factors (dietary supplements, dietary diversity, breastfeeding, and YYB intervention) as potential covariates. Caregiver-level factors comprised parent–child care, educational level, and economic status. The chief guardians were categorized into two groups—mother or father and non-parents. The household socioeconomic status (SES) is a representative measure of environmental variables [26]. The WHO defines children’s dietary diversity, a key indicator for assessing their supplementary feeding status, as the number of different food groups consumed before the age of 6 [27]. This was determined through mothers’ recollections of all foods and beverages provided to each child within 24 h prior to the survey. Data on the consumption frequency of seven categories of complementary foods were gathered: grains, roots, and tubers; vitamin A-rich fruits and vegetables; other fruits and vegetables; fresh foods (such as meat, fish, poultry, and organ meats); eggs; dairy products (including milk, infant formula, yogurt, and cheese); and legumes and nuts. Dietary supplements, breastfeeding, and the YYB intervention were divided into two groups —yes and no.

### 2.4. Data Analysis

The Kolmogorov–Smirnov test was used to evaluate the normality of continuous outcome variables. Overview statistics, encompassing mean values ± SDs and counts (percent figures), were utilized to summarize the cohort participant’s characteristics. For continuous variables, paired *t*-tests were utilized, while categorical variables were evaluated via chi-square tests. Given the repeated measurements taken from the children, this study used a mixed linear regression model to assess the neurodevelopment of 5-year-old children and their conditional height/weight data from ages 1 to 5. Furthermore, a multivariate linear regression model was applied to evaluate the neurodevelopment of 5-year-olds and the WAZ/LAZ/BAZ across various age groups. These associations were illustrated using forest plots. Two models were used to examine associations with each outcome: one was adjusted solely for the child’s age and gender, and another completely accounted for child-specific factors (age, gender, birth weight, birth length, and birth outcomes), caregiver (parent–child care and education level), caregiving practices, and environmental factors (SES). Results were shown as changes in outcomes related to a 1 SD shift in a child’s dimensions between ages 1 and 5 or in conditional variables. All reported *p* values were two-tailed, with differences deemed statistically significant when *p* < 0.05. EpiData (version 3.1; EpiData, Odense, Denmark) was used to establish a database and conduct double-entry verification to exclude the data of individuals with missing key information or outside the mean ± 5 SDs range. R (Version 4.1.2, “MatchIt” package) was used for PSM, and GraphPad Prism software (Version 9.0) was used for the forest plots. For other statistical analyses, STATA (Version 16.0, StataCorp, College Station, TX, USA) was employed.

## 3. Results

### 3.1. Primary Characteristics of Participants

We successfully followed up with 95.74% of the LHEAPITNP cohort (*n* = 1057 out of 1104 children), comprising children aged ≤ 6 years with available data from 2018 to 2022 on anthropometry and neurodevelopment (Figure 1). The primary cause of data gaps was the absence of participants during visits, which occurred due to illness, family relocation, or caregivers being unable to enroll children in community health centers over the course of the study. The groups (YYB-CG and YYB-IG) showed a similar rate of children with missing data. Nonetheless, there were baseline differences in caregiver and dietary environment characteristics by the groups. PSM was used to eliminate these feature differences between the groups for the final analytic sample (Table 1). After PSM, there were 474 pairs of children, and the enrolled children were 1.27 ± 0.42 years old in the YYB-CG and 1.27 ± 0.41 years old in the YYB-IG, with nearly half of them being girls in each group. Data for unpaired samples in both groups were not used in subsequent analyses.

### 3.2. Nutritional Condition of Subjects Affected by YYB

**Figure 2:**
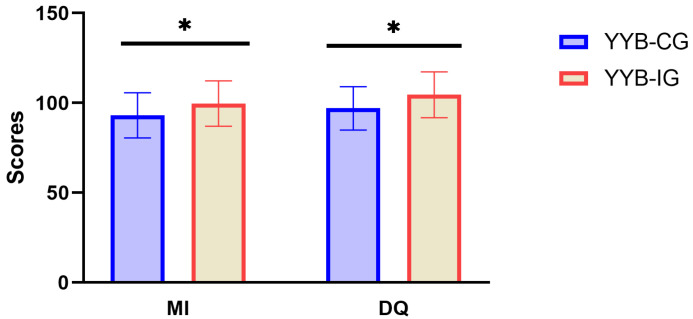
Neurodevelopment for children in 2022 in YYB-CG and YYB-IG. YYB-IG: Ying Yang Bao intervention group; YYB-CG: Ying Yang Bao control group; MI: mental index; DQ: developmental quotient. * indicates *p* < 0.05.

As shown in Figure 2, children in the YYB-IG group had significantly higher MI (99.73 ± 12.62) and DQ (104.55 ± 12.77) scores in 2022 than those in the YYB-CG group, which had an MI of 93.11 ± 12.58 and DQ of 97.02 ± 12.04 (*p* < 0.001).

The descriptive statistics for children’s attained body dimensions and nutritional well-being are shown in Table 2. A slightly lower height for 1-year-old children was observed for the YYB-IG compared to the YYB-CG, but the situation was exactly the opposite by age 5 (*p* = 0.015). The YYB-IG had a significantly higher BAZ compared to the YYB-CG both at 1 year and at 5 years old (*p* values: 0.013 and 0.007, respectively), with the disparity in BAZ being slightly greater at 5 years than at 1 year old. Similar rates of being underweight were observed in the YYB-IG compared to the YYB-CG for children of 1 year (*p* = 0.834), whereas at 5 years old, the YYB-IG showed a smaller decrease in the risk of being underweight (*p* = 0.015). At 1 year old, the rate of stunting children was comparable between the YYB-IG and YYB-CG (*p* = 0.300), but at age 5, the decline in stunting risk for the YYB-IG was relatively modest (*p* = 0.040). The groups showed no notable variation in weight, WAZ, HAZ, or stunting (*p* > 0.05).

### 3.3. Correlations Between Growth and Neurodevelopment Among Preschoolers

**Figure 3:**
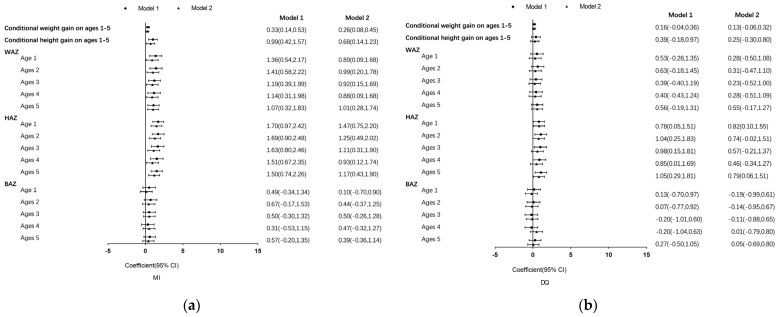
(**a**) Associations between child growth and MI score are contained in the first panel; (**b**) associations between child growth and the DQ is contained in the second panel. Model 1 was modified to account for age and gender of the child; model 2 was calibrated for individual child factors (gender, age, weight at birth, length at birth, and birth-related outcomes), caregiver (parent–child care, education level, and the YYB intervention), caregiving practices, and environmental factors (SES). MI, mental index; DQ, developmental quotient; WAZ, Z-score for weight relative to age; HAZ, height for age Z-score; BAZ, body mass index for age Z-score.

Figure 3 shows the association between the various developmental subdomains and overall neurodevelopment at age 5. Faster weight gain between ages 1 and 5 was linked to an increased MI score (β (95% CI): 0.26 (0.08–0.45)). Weight gain during ages 1 to 5 exhibited a positive relationship with the DQ (β (95% CI): 0.13 (−0.06–0.32)), but this correlation lacked statistical significance once confounding factors were controlled. Correspondingly, faster height improvement during this timeframe was tied to a higher MI score (β (95% CI): 0.68 (0.14–1.23)). Rapid height gain was associated with a higher DQ (β (95% CI): 0.25 (−0.30–0.80)), yet the observed differences lacked statistical significance. Higher WAZs were associated with higher MI scores at 1 year (β (95% CI): 0.89 (0.09–1.68)), 2 years (β (95% CI): 0.99 (0.20–1.78)), 3 years, (β (95% CI): 0.92 (0.15–1.69)), 4 years (β (95% CI): 0.88 (0.09–1.68)), and 5 years old (β (95% CI): 1.01 (0.28–1.74)). The WAZ also showed a positive association with the DQ, although the differences were not statistically significant. A higher HAZ was associated with a higher MI score at 1 year (β (95% CI): 1.47 (0.75–2.20)), 2 years (β (95% CI): 1.25 (0.49–2.02)), 3 years (β (95% CI): 1.11 (0.31–1.90)), 4 years (β(95% CI): 0.93 (0.12–1.74)), and 5 years old (β (95% CI): 1.17 (0.43–1.90)). The HAZ was also associated with a higher DQ at 1 year (β (95% CI): 0.82 (0.10–1.55)) and at 5 years old (β (95% CI): 0.79 (0.06–1.51)). While the BAZ during the preschool period was positively associated with the MI score, the difference did not reach statistical significance, and there was no link to the DQ.

## 4. Discussion

Early malnutrition is related to developmental delays during the formative years, along with reduced cognitive performance and weaker achievement and troubleshooting abilities later in life. Stunting, a measure of delayed growth that affects over 30% of children under 5 years old [28], is linked to cognitive delays between ages 5 and 11 [29], reduced academic achievement, and a higher risk of school dropout [30]. Analogously, findings from a study in Burkina Faso suggest that poor linear growth in childhood is related to underdeveloped communication and movement skills at 18 months [31]. A meta-analysis involving observational research in low- and middle-income countries (LMICs) projected that a 1 SD increase in HAZ was related to a 0.28 SD rise in cognitive scores and a 0.24 SD rise pertaining to motor growth scores in children below age 2 [32]. During the preschool stage, an essential period marked by significant and rapid alterations in neural adaptability occurs, leading to the acquisition of essential cognitive and social [33]. In a birth cohort study conducted in New Delhi, India, height growth between birth and six months and from six months up to age two, as well as BMI growth spanning six months to the age of two, were linked favorably to cognitive abilities; body growth measurements accounted for 8% of educational attainment as an indicator of cognitive ability [14]. In a multicenter, open, prospective randomized trial involving six Italian centers, Esposito et al. found that the sole indicator for a greater risk of suboptimal intelligence quotient (IQ) at ages 1 to 2 was a delayed bone age at diagnosis [34]. In a Pediatric Heart Network trial conducted in North America, a low-height trajectory was associated with worse neurodevelopment [35].

This study demonstrates the prolonged influence of YYB in preschool years on children’s growth and neurodevelopment. YYB is rich in high-quality proteins and vitamins like A, B, C, and D, along with essential minerals (including calcium, iron, zinc, and folate), with nutrient ratios that meet children’s needs [6]. The proteins in YYB, derived from milk or soybeans, effectively support physical growth and cognitive development [13]. Vitamin D and calcium in YYB contribute significantly to promoting skeletal growth [36]. Iron [37], zinc [38], and folic acid [39] can individually or collectively support the production of hormones, enzymes, and other substances vital for brain development. Mineral absorption, including iron, calcium, and zinc, is promoted by vitamins D and C [40]. The WHO identified YYB as a cost-efficient strategy to enhance children’s nutrition in resource-limited areas [41]. Additionally, these findings align with additional research that investigated the prolonged influence of preschool nutritional interventions. To illustrate, 2-year nutritional supplementation of North American children aged 9 to 24 months with developmental delays positively influenced their intelligence, education level, and knowledge by age 22 [42]. A nutritional intervention trial conducted involving Iranian children aged 24 to 59 months showed that an 8-week supplementation with ready-made foods significantly improved weight, height, and BMI [43]. Early life is a critical window for developing human capital and there is a rising understanding of how crucial early life interventions are, as a child’s nutritional status during this period may influence their lifelong health and achievement. In addition, this study observed slight differences in neurodevelopment at 5 years of age. While these effects may be due to chance, the ability to detect such differences may improve with age as children reach new developmental milestones. It is also possible that higher-level intellectual functions, such as language and abstract reasoning based on visual abilities, remain latent and only manifests later, when children face more cognitively demanding environments [44]. Ongoing monitoring of this group of Chinese children is essential to determine if the effects of YYB on growth and development persist and increase with age.

Besides the findings discussed, this study also expands on previous findings by confirming earlier reports of the beneficial relationships between linear growth in children during early school ages and later neurodevelopment [14,34]. We divided the period before age 6 into five time points, encompassing ages 1, 2, 3, 4, and 5 years. Faster height gain from 1 year to 5 years old was associated with higher scores in total neurodevelopment, as measured by the MI. Although the coefficients for linear growth from 1 year to 5 years, with respect to MI subdomains, were consistently greater and estimated with more accuracy than those for WAZ or HAZ between ages 1 and 5, we are unable to determine that the strength of the connections varied significantly due to substantial overlap in the CIs. In the same way, the coefficients for linear growth from 1 year to 5 years, with respect to the DQ subdomains, were reliably greater and assessed with higher accuracy than those for HAZs between ages 1 and 5.

Some studies indicate that growth during the first year has a stronger connection with neurodevelopment compared to growth in later years until early schooling. For instance, research conducted in Thailand indicated that growth during the first year was related to neurodevelopment at 9 years, while growth from ages 1 to 9 was not [45]. An analysis of six forward-looking birth groups from five LMICs—South Africa, Guatemala, Brazil, the Philippines, and India—revealed that linear growth near age 2 was a predictor of IQ and academic achievement in adulthood, whereas linear growth during subsequent stages was not linked to these results [30]. Additionally, the Infant Health and Development Program cohort study suggested that modest neurodevelopmental benefits were associated with a swifter increase in weight during the initial year: each increase in WAZ corresponded to a 1.9-point increase in intelligence scores [46]. In contrast, our study indicates that growth from 1 year through early education plays a key role in neurodevelopment. In a large cohort study conducted in Vietnam and Asia, BAZ was not associated with intellectual function in 2-year-olds. However, accelerated growth in height from ages 2 to 7 years were linked to enhanced cognitive function [47]. In an Asian Filipino cohort, shifts in the HAZ from 6 months to 2 years were tied to greater increases in cognitive abilities than from 2 months to 11 years [48]. The Young Lives Study, conducted in Peru, India, and Ethiopia, has noted analogous findings [49,50,51]. Both the HAZ at approximately 1 year and contextual growth from ages 1 to 8 exhibited positive connections with vocabulary comprehension, mathematical proficiency, and reading skills [50]. Moreover, kids who regain linear growth between ages 1 and 8 also typically perform better than those who remain stunted during this timeframe [45]. Nonetheless, these analyses contain a restriction in that the evaluations of subsequent growth included considerable growth before age 2, as the assessment representing this period was taken between 6 and 18 months of life.

This study has several methodological limitations. Despite adjusting for a broad spectrum of child factors, unaccounted confounding due to hereditary and situational elements remains a possibility. The four accounted dietary factors (dietary supplements, dietary diversity, breastfeeding, and YYB intervention) are appropriate for assessing feeding practices during the preschool years, particularly during the transition from breastfeeding to complementary feeding (before the age of 3). However, a single 24-hour dietary recall was used to assess child feeding, and this method may not accurately reflect overall dietary exposure. Additionally, the age of introduction of complementary foods and food availability status were not included in our questionnaire. Attrition caused by relocation or disinterest may have also led to a nonresponse bias. Nevertheless, given that the groups were consistent in various initial traits, we anticipate that unobserved characteristics will be similarly balanced.

The major strength of this research is its reliance on prospectively gathered information from a well-established cohort of children spanning 1 to 5 years old with a strong follow-up record, combined with comprehensive measures of neurodevelopment and its subdomains. Every assessment instrument underwent validation and was appropriately tailored to the local context [18]. The longitudinal analysis accounted for missing data and assessed the impact of observations over time. Additionally, the analytical method, employing conditional growth metrics, minimized collinearity between multiple measurements and allowed for a distinction between linear growth and soft tissue gain [21].

## 5. Conclusions

Linear growth during preschool years (before age 6) is positively associated with neurodevelopment in children aged 5. While early interventions within the first 2 years remain crucial, it is essential to consider appropriate interventions at various stages throughout the life course, including the preschool stage. Additionally, our study examined the preschool phase and analyzed the weekly outcomes of YYB intake during this period and its long-term impacts on children’s physical and neurological development. The results of this trial support YYB as a possibly beneficial strategy for enhancing children’s growth and neurodevelopment in later life. The findings provide valuable, novel data in these areas. Future research should investigate whether growth during the school-age period can effectively predict neurodevelopment, and if the impacts of early YYB on children’s growth and neurodevelopment persist into the school-age period.

## Figures and Tables

**Table 1:** Description of study children within the initial and paired datasets.

	Initial			Paired		
	YYB-CG (*n* = 528)	YYB-IG (*n* = 529)	*p* Value	YYB-CG (*n* = 474)	YYB-IG (*n* = 474)	*p* Value
Age, years	1.26 ± 0.44	1.28 ± 0.41	0.486	1.27 ± 0.42	1.27 ± 0.41	0.859
Gender, *n* (%)			0.214			0.948
Male	328 (53.2%)	318 (49.7%)		244 (51.5%)	242 (51.1%)	
Female	288 (46.8%)	322 (50.3%)		230 48.5%)	232 (48.9%)	
Birth weight, g	3.26 ± 0.46	3.24 ± 0.45	0.354	3.26 ± 0.48	3.25 ± 0.45	0.888
Birth height, cm	50.03 ± 1.76	49.99 ± 1.29	0.595	50.07 ± 1.74	50.02 ± 1.31	0.635
Preterm, *n* (%)	21 (3.4%)	19 (3.0%)	0.748	18 (3.8%)	15 (3.2%)	0.724
Chief guardian, *n* (%)			0.015			0.655
mother or father	486 (78.9%)	467 (73.0%)		357 (75.3%)	350 (73.8%)	
non-parents	130 (21.1%)	173 (27.0%)		117 (24.7%)	124 (26.2%)	
Chief guardian’s education, *n* (%)			0.020			0.310
Below primary level	175 (28.4%)	162 (25.3%)		120 (25.3%)	129 (27.2%)	
Secondary school	332 (53.9%)	324 (50.6%)		245 (51.7%)	255 (53.8%)	
High school level or greater	109 (17.7%)	154 (24.1%)		109 (23.0%)	90 (19.0%)	
Consume other supplements, *n* (%)	161 (26.1%)	101 (15.8%)	<0.001	65 (13.7%)	76 (16.0%)	0.361
Dietary diversity, score	3.97 ± 1.67	4.78 ± 1.66	<0.001	4.48 ± 1.53	4.59 ± 1.65	0.286
SES, score	54.14 ± 4.85	54.67 ± 5.09	0.093	54.61 ± 4.69	54.09 ± 4.90	0.097

Values are mean values ± SDs or counts (percent figures). SES: socioeconomic status; YYB-CG: Ying Yang Bao control group; YYB-IG: Ying Yang Bao intervention group.

**Table 2:** Summary statistics of the children in the matched samples with their neurodevelopmental outcomes and their nutritional statuses.

	YYB-CG (*n* = 474)	YYB-IG (*n* = 474)	*p* Value
Weight, kg			
Age 1	9.58 ± 1.52	9.69 ± 1.47	0.252
Ages 2	11.66 ± 1.70	11.80 ± 1.66	0.186
Ages 3	13.96 ± 2.11	13.85 ± 1.97	0.427
Ages 4	15.75 ± 2.37	15.76 ± 2.30	1.000
Ages 5	17.78 ± 3.02	17.92 ± 3.08	0.500
Length, cm			
Age 1	76.66 ± 6.06	76.48 ± 5.84	0.642
Ages 2	86.47 ± 5.23	86.62 ± 4.96	0.664
Ages 3	95.33 ± 5.21	95.53 ± 4.78	0.556
Ages 4	102.00 ± 5.21	102.52 ± 5.03	0.126
Ages 5	108.69 ± 5.54	109.57 ± 5.36	0.015 *
WAZ			
Age 1	−0.27 ± 0.96	−0.17 ± 1.06	0.148
Ages 2	−0.39 ± 1.01	−0.31 ± 1.00	0.218
Ages 3	−0.36 ± 1.03	−0.41 ± 1.03	0.414
Ages 4	−0.36 ± 0.98	−0.40 ± 1.00	0.522
Ages 5	−0.36 ± 1.10	−0.34 ± 1.09	0.733
HAZ			
Age 1	−0.39 ± 1.19	−0.41 ± 1.10	0.466
Ages 2	−0.43 ± 1.13	−0.41 ± 0.96	0.218
Ages 3	−0.39 ± 1.04	−0.34 ± 0.95	0.405
Ages 4	−0.35 ± 0.99	−0.30 ± 0.98	0.431
Ages 5	−0.29 ± 1.23	−0.16 ± 0.96	0.061
BAZ			
Age 1	−0.10 ± 0.95	0.05 ± 1.01	0.013 *
Ages 2	−0.25 ± 0.93	−0.15 ± 1.00	0.147
Ages 3	−0.20 ± 1.02	−0.33 ± 1.02	0.055
Ages 4	−0.24 ± 0.99	−0.35 ± 0.99	0.075
Ages 5	−0.44 ± 1.04	−0.25 ± 1.0	0.007 *
Underweight			
Age 1	10 (2.5%)	13 (2.3%)	0.834
Ages 2	22 (5.6%)	14 (2.5%)	0.024 *
Ages 3	14 (3.6%)	30 (5.4%)	0.211
Ages 4	11 (2.8%)	20 (3.6%)	0.580
Ages 5	17 (4.3%)	22 (4.0%)	0.869
Stunting			
Age 1	31 (7.9%)	34 (6.1%)	0.300
Ages 2	24 (6.1%)	19 (3.4%)	0.058
Ages 3	21 (5.3%)	15 (2.7%)	0.040 *
Ages 4	17 (4.3%)	19 (3.4%)	0.495
Ages 5	18 (4.6%)	14 (2.5%)	0.101
Wasting			
Age 1	9 (2.3%)	14 (2.5%)	1.000
Ages 2	13 (3.3%)	9 (1.6%)	0.124
Ages 3	15 (3.8%)	23 (4.2%)	0.867
Ages 4	10 (2.5%)	13 (2.3%)	0.834
Ages 5	15 (3.8%)	21 (3.8%)	1.000

Values are mean values ± SDs or counts (percent figures). WAZ, Z-score for weight relative to age; HAZ, Z-score for height relative to age; BAZ, Z-score for body mass index by age. * indicates *p* < 0.05.

## Data Availability

The datasets generated and analyzed during the current study are not publicly available, but are available from the corresponding author on reasonable request.
